# Nursing care needs and services utilised by home-dwelling elderly with complex health problems: observational study

**DOI:** 10.1186/s12913-017-2600-x

**Published:** 2017-09-12

**Authors:** Gro Næss, Marit Kirkevold, Wenche Hammer, Jørund Straand, Torgeir Bruun Wyller

**Affiliations:** 1 CHARM Research Centre for Habilitation and Rehabilitation Models & Services, Institute of Health and Society, University of Oslo, Oslo, Norway; 2 Department of Nursing Science, Institute of Health and Society, University of Oslo, Oslo, Norway; 3 Department of Health and Caring, Eidsberg Municipality, NO-1850 Mysen, Norway; 4 Department of General Practice, Institute of Health and Society, University of Oslo, Oslo, Norway; 5 Institute of Clinical Medicine, University of Oslo, Oslo, Norway; 6 Department of Geriatric Medicine, Oslo University Hospital, Oslo, Norway

**Keywords:** Elderly, Community care, Home nursing, Frailty, Multimorbidity, Polypharmacy, Functional decline

## Abstract

**Background:**

In Norway, as in many Western countries, a shift from institutional care to home care is taking place. Our knowledge is limited regarding which needs for nursing interventions patients being cared for in their home have, and how they are met. We aimed at assessing aspects of health and function in a representative sample of the most vulnerable home-dwelling elderly, to identify their needs for nursing interventions and how these needs were met.

**Methods:**

In this observational study we included patients aged 75+ living in their own homes in Oslo, who received daily home care, had three or more chronic diagnoses, received daily medication, and had been hospitalized during the last year. Focused attention and cognitive processing speed were assessed with the Trail Making Test A (TMT-A), handgrip strength was used as a measure of sarcopenia, mobility was assessed with the “Timed Up-and-Go” test, and independence in primary activities of daily living by the Barthel Index. Diagnoses and medication were collected from electronic medical records. For each diagnosis, medication and functional impairment, a consensus group defined which nursing service that the particular condition necessitated. We then assessed whether these needs were fulfilled for each participant.

**Results:**

Of 150 eligible patients, 83 were included (mean age 87 years, 25% men). They had on average 6 diagnoses and used 9 daily medications. Of the 83 patients, 61 (75%) had grip strength indicating sarcopenia, 27 (33%) impaired mobility, and 69 (83%) an impaired TMT-A score. Median amount of home nursing per week was 3.6 h (interquartile range 2.6 to 23.4). Fulfilment of pre-specified needs was >60% for skin and wound care in patients with skin diseases, observation of blood glucose in patients taking antidiabetic drugs, and in supporting food intake in patients with eating difficulties. Most other needs as defined by the consensus group were fulfilled in <10% of the patients.

**Conclusions:**

We identified a very frail group of home-dwelling patients. For this group, resources for home nursing should probably be used in a more flexible and pro-active way to aim for preserving functional status, minimize symptom burden, and prevent avoidable hospitalisations.

## Background

Frail older people who receive health care in their home often have needs for comprehensive health care [1]. Many suffer from an unstable health characterized by several chronic diseases, functional impairments and polypharmacy [2]. They are at high risk for developing additional functional decline, and may be particularly good candidates for preventive and early interventions [3]. In this patient group, symptoms of new diseases or worsening of existing diseases may be vague and uncharacteristic. However, prompt diagnosis and treatment of acute or subacute changes in health state, combined with adequate rehabilitation measures may prevent further functional decline and hospitalization [4–7], and give long-term benefits by maintaining the care recipients’ functional status and quality of life [8–10]. In a recent review, Philip and co-workers reported that many hospital-based as well as community-based interventions developed may prevent hospital admittance of frail elderly persons [11].

Despite the fact that patients most of the time receive treatment and care in the communities, there is limited research data regarding the needs and follow-up care of older patients living at home. Community care is becoming increasingly important across a number of countries, and one of its aims is to prevent unnecessary hospital admissions [12]. In Norway, hospitals are run by the national government and nursing homes and home care services by the municipalities or by private companies working on a contract with the municipality. This “two-track system”, in combination with shortage of hospital beds, constitutes an incitement for hospitals to discharge patients early, pushing more responsibility for frail patients on the local communities. The Coordination Reform, implemented from 2012, transfers responsibility from hospitals to the municipal health and care services. Many of the municipalities, including the capital of Oslo have been organised with one authority responsible for ordering services and another responsible for providing them. A list system ensures all inhabitants their own general practitioner (GP). The list holding GPs have a contract with their municipality and most practices are organized as independent enterprises with a combination of public funding and fee for service. The Research Council of Norway, on assignment for the Ministry of Health and Care Services, have completed an evaluation of the health and care services after implementation of the Norwegian Coordination Reform. The evaluation reveals that there is a lack of knowledge and evidence-based guidelines for older patients with multimorbidity. This is a large patient group with extensive need of healthcare ([13], p. 51). Internationally, frequent readmissions are common in frail, home-dwelling elderly, and may also represent a burden and a risk factor for further functional decline [14]. In order to tailor the community care services to the needs of frail patients, precise knowledge about their health and functional status is needed. Little is known, however, about the functional characteristics and trajectories of seniors receiving nursing care in their own home, their needs for qualified health care, and how these needs are met. Better knowledge on this is a prerequisite for improving the services when necessary.

Cognitive dysfunction, sarcopenia, impaired mobility, dependency in activities of daily living (ADL), multimorbidity and polypharmacy are core elements of frailty [15]. Frailty is a major risk factor for rapid health deterioration and poor health outcomes. Thus identifying frailty among older individuals is essential to initiate preventive and health promoting measures as early as possible [16]. Indicators reflecting these core elements are therefore helpful in targeting seniors in need of intensified surveillance and multiprofessional follow-up.

Accordingly, the aim of this study was to describe the needs for nursing assessment and care in a Norwegian cohort of frail, home-dwelling elderly persons receiving home care, and to evaluate whether the currently provided nursing care adequately addressed their needs.

## Methods

### Study design

This study had a cross-sectional design with an eight months follow-up. A structured evaluation was undertaken on the appropriateness of the care provided. For this evaluation, we utilised consensus-based criteria developed by a multi-professional expert team for this study. The team defined what should be regarded as a minimal justifiable level of nursing care in relation to defined health states (described below).

### Setting and study population

The study was carried out in three of the 15 administrative districts in Oslo, Norway, with a total population of about 144,000 (out of 619,000 in Oslo). The socioeconomic status in the three districts roughly equals Oslo in total. With respect to demographics, the age group 67–79 years is as in the total city (7.4% versus 7.0%), whereas the percentage aged 80+ is somewhat higher (4.7% versus 3.5%). The districts are responsible for providing home nursing care for all inhabitants in need for this. The care is based upon formal assessments and decisions by a local administrative unit designated for this purpose. These decisions also include the number of minutes per week of home nursing care each patient is allotted.

We aimed at studying the frailest among elderly home dwelling people, and patients were eligible for inclusion if the following criteria were met:

- Age 75 years or more
- Home-dwelling
- Receiving home care on a daily basis
- Three or more chronic diagnoses recorded in the home nursing service’s patient record, and
- Hospitalized at least once during the last year.

Exclusion criteria were terminal illness, and severe dementia making informed consent impossible (as evaluated by the nurse in charge for their care).

Homecare nurses in the districts identified eligible patients and obtained written, informed consent for study participation.

### Data collection

Data were collected during the period from January 2013 to February 2014. The head nurses for the respective districts identified patients that fulfilled the inclusion criteria. They were asked to omit patients that had one of the exclusion criteria described above. The head nurses had been explained the aim of the study and the consequences of participating. The patients then got oral and written information, and gave their written consent to participate. Three registered nurses specially trained in assessing elderly patients, as well as one medical student, carried out the clinical assessments of cognitive functioning, sarcopenia, independence in activities of daily living (ADL), and mobility in the participants’ homes at baseline and after eight months. At both visits, the patients were asked if they knew the names and indications of their medicines.

One nurse from each district extracted data from the nursing districts’ electronic records on demographics, chronic diseases and medication, as well as information about the content of the service provided for each individual participant. Nursing documentation included nursing care plans, medication charts, progress notes and summaries. Emphasis is increasingly put on formal documentation in the caring services, making it possible to extract information from the case notes on which evaluations have been done. Since information was sampled from several documents, we manually reviewed data for consistency and duplications.

### Instruments

As indicators of the patients’ need for nursing services, we recorded diagnoses, current drug treatment, cognitive functioning, sarcopenia, mobility, and independence in ADL.

For assessment of aspects of cognitive functioning, we employed the Trail Making Test (TMT) A, which is sensitive for focused attention and cognitive processing speed. It has no ceiling effects and is considered to be a sensitive indicator of a patient’s general condition. If the patient uses more than 60 s on the task, it is generally perceived as a sign of impaired focused attention and/or reduced psychomotor speed [17].

Handgrip strength was used as a measure of sarcopenia. Hand grip was measured with a Jamar® hydraulic hand dynamometer type 5030 J1 (Jackson, MI, USA) with adjustable handgrip. The participants were sitting with the arm resting and the dynamometer in an upright position, and the handgrip strength was registered as maximum kilograms of force during a trial. The mean of three trails (for each hand) was used. The measurement was not performed if the subject had a current hand pain. Handgrip strength below 30 Kg in men and below 20 Kg in women was considered indicative of sarcopenia [18].

Mobility was assessed with the “Timed Up-and-Go” test (TUG). TUG measures the time needed to stand up from a chair, walk three meters, turn around, return and sit down again. Three trails were performed, and we used the mean result in the analysis. More than 30 s used to carry out TUG suggests a mobility problem [19]. TUG was not performed if the patient had severe pain while walking or needed support of another person to be able to walk.

Independence in primary ADL tasks was assessed by the Barthel ADL Index, a widely used and well validated instrument [20]. The Barthel Index was filled in by the project nurses.

### Quality criteria for appropriate nursing care

To define elements of health services needed for adequate care delivery for elderly and frail patients, a multi-professional expert team was established for this study. The panel comprised one nurse practitioner in geriatric nursing, working in primary care (in another municipality than the one studied) (WH), one nurse researcher teaching advanced geriatric nursing (GN), one professor in general practice (JS), and one professor in geriatric medicine (TBW). The team defined what should be regarded as a minimal justifiable level of nursing care in relation to the recorded cognitive functioning, muscle function, mobility, ADL functioning, diagnoses and drug use. Initially, the first author (GN) suggested observations and interventions deemed necessary for each diagnosis, drug class or functional limitation. The suggestions were reviewed by the other panellists independently of each other. Only interventions that were deemed necessary by all four panellists were kept in the final list. We thus applied a conservative approach, ensuring that only observations and interventions that were considered as necessary by all four panel members were used as a reference standard for the services that were recorded in relation to the listed challenges. The consensus panel did only define a need if they considered the particular diagnosis, medication use or functional limitation as necessitating specific, qualified nursing interventions or observations.

### Analysis

Based on all available information, we evaluated whether or not health service elements deemed necessary for appropriate care were in fact delivered to the patient. Descriptive statistics was carried out using SPSS version 2.0.

### Ethics

Ethics approval was obtained from the Regional Committee for Medical and Health Research Ethics (REK). District nurses obtained written informed consent from all patients participating in the study. The patients were explained that the aim of the study was to assess the needs of elderly home-dwelling persons receiving nursing services in their homes. Patients unable to provide an informed consent were not included. Patients were assured that participation in the study did not impact on their health care. The nurses carrying out the assessments were instructed to secure the participants’ well-being during the interview process and to interrupt the interview if the patient showed any sign of discomfort. The participants were considered to be exposed to negligible risk due to the study. Written information was stored in closed and fire-safe cabinets, and digital data at secured data disks at the University of Oslo.

## Results

**Fig. 1:**
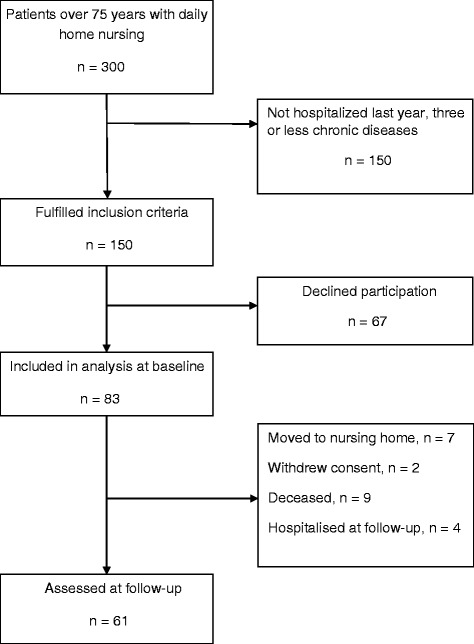
Patient flow

Among the home nursing clients in the three districts a total of 150 eligible patients were identified. Of these, 83 gave informed consent and were included. During the next eight months, further 22 patients dropped out, leaving 61 patients for the follow-up assessment (Fig. 1). Descriptive statistics are provided in Table 1. At baseline, the patients were on average 87 years, three in four were women, and nearly four in five lived alone. They had more than five diagnoses requiring active treatment and used nearly nine daily medications. Most conditions were related to cardiovascular disease. Non-cardiovascular conditions such as osteoarthritis, osteoporosis and fractures were also common, and often resulted in pain as a major health problem. A high fraction of the patients had ADL limitations. 60% of the patients did not themselves know which medications they used.

**Table 1:** Descriptive variables and functional changes at baseline and at after eight months

Variable	Baseline (*n* = 83 if not otherwise stated)	Baseline values in patients with a follow-up value	Follow up (*n* = 61 if not otherwise stated)	Mean change (95% CI)
Age, years; mean (SD)	87 (4.4)			
Male gender; n (%)	21 (25)			
Living alone; n (%)	65 (78)			
Chronic conditions receiving active treatment; mean (SD)	6 (3.6)			
Daily medications; mean (SD)	9 (4.2)			
Barthel ADL-index score; median (IQR)	12 (10–14) *n* = 72			
Handgrip strength, Kg; mean (SD)	17 (8.8) *n* = 81	17 (9.2)	17 (8.6) n = 61	−0.6 (−1.5, 0.2)
TUG, Seconds; mean (SD)	30 (19.2) *n* = 75	27 (12.2)	31 (15.4) *n* = 52	4.6 (2.2, 7.0)
TMT-A, minutes; median (IQR)	95 (67–164) *n* = 82	94 (66–155)	105 (80–153) n = 61	8.7 (−13.4, 18.6)
Weight, Kg; mean (SD)	69 (16.2) *n* = 80	65 (14.9)	65 (14.2) *n* = 57	0.2 (−1.2, 1.6)

*TUG* Timed Up-and-Go, *TMT* Trail Making Test, *ADL* Activities of Daily Living, *IQR* Interquartile range

At baseline, 61 of 81 patients (75%) had a grip strength indicating sarcopenia, increasing to of 50 of 61 (82%) at follow up. Regarding mobility, 27/83 patients (33%) were impaired at baseline and 26/61 (43%) at follow up, whereas the fraction of patients with a TMT-A score indicating impaired attention and/or reduced psychomotor function remained stable (69/83 [83%] at baseline versus 51/61 [84%] at follow-up).

Median amount of home nursing per week according to the formal decisions was 3.6 h (interquartile range 2.6 to 23.4). That is, 31 min a day, most often distributed in two or three visits. Services provided by the community are summarised in Table 2. The most common nursing services were assistance with medications (nearly all patients) and personal hygiene (four in five), followed by specific nursing tasks (such as care of urinary catheter, stoma care, bladder irrigation, blood glucose testing). Help with medications was regularly defined in the patient records as “delivering prescribed medications to the patient”, but observation of effects or adverse effects were not noted. Most of the patients got their medication by a multidose drug dispensing system, and in these cases information regarding the indication for the different drugs was rarely available in the nursing documentation system. More than half the patients were found to be in need of a personal body worn security alarm due to an increased risk of falling, but less than one in ten received physiotherapy and/or occupational therapy. Needs for rehabilitation interventions to strengthen self-care or to prevent falls were not reported in any of the patient records. Help with compression stockings was explicitly limited to assisting in taking the stockings on and off, not including assessment of oedema, skin changes or possibly related pain.

**Table 2:** Service provided by community. *n* = 83

Type of service	N (%)
Components of home nursing	
Medication management	81 (98)
Personal hygiene assistance	68 (82)
Specific nursing procedures (e.g. maintenance of urinary catheter, stoma, tube feeding)	43 (52)
Nutritional support	42 (51)
Compression stocking application	21 (26)
Observation (e.g. blood pressure, weight)	19 (23)
Wound care	9 (11)
Other services	
Personal transportation service	53 (64)
Security alarm	46 (55)
Home help (e.g. housecleaning)	44 (53)
Day centre care	15 (18)
Physiotherapy	7 (8)
Occupational therapy	7 (8)
Meals on wheels	4 (5)

Table 3 displays the most common chronic conditions the patients suffered from, the main groups of medication used, and the number of patients with the various functional limitations that were evaluated. For each disorder, drug class or functional limitation, the table also displays regular nursing interventions or observations needed for the condition according to the expert panel, and the number of patients with the particular condition that actually had received the recommended intervention. Fulfilment of needs was high for skin and wound care, observation of blood glucose in patients on antidiabetics, and in supporting food intake in those with eating difficulties, but a vast majority of the defined needs identified by the expert panel was fulfilled in less than one in ten patients.

**Table 3:** Diseases under active treatment, medication, functional status, derived needs (as defined by consensus group), and services utilised. *n* = 83

Condition	n (% of 83)	Needs	n (% of patients with condition) utilising relevant nursing care according to electronic nursing records^a^
Functional limitations			
Mobility limitation. TUG >30 s	28 (34)	Observe and encourage walking	4 (14)
		Secure patient when walking	5 (18)
		Observe dizziness, unsteadiness and falls	4 (14)
		Observe and treat pain related to walking	0
Sarcopenia. Grip strength <30 kg (males) or <20 kg (females)	61 (75)	Observe signs of declining muscle strength when carrying out ADL tasks	0
		Encourage activity and stimulate simple exercising	1 (1)
		Initiate fall prevention	2 (3)
Need of assistance for nutrition	32 (39)	Support food intake	20 (63)
		Assess nutrition and problems with food intake	12 (38)
		Observe gastrointestinal symptoms and weight change	5 (16)
Serious disability. Barthel ADL-index score < 11	36 (43)	Observe changes in ADL-function	3 (8)
		Encourage self-care and means to improve ADL	2 (6)
Possibly impaired cognition. TMT-A > 60 s	69 (83)	Observe changes in cognitive function	2 (3)
		Identify sensory impairments	2 (3)
Diseases			
Cardiovascular	58 (70)	Monitor pulse and blood pressure	5 (9)
		Observe skin and hydration	5 (9)
		Observe and treat oedema, dyspnoea and cough	3 (5)
Osteoporosis and fractures	28 (34)	Observe signs of new fractures	0
		Identify risk of falling	3 (11)
		Observe changes in pain and joint function	0
Osteoarthritis of hip or knee	15 (18)	Observe and encourage activity	0
		Identify needs of assistive aids	0
		Observe changes in pain	1 (7)
Stomach and intestinal disease	21 (25)	Observe nutrition, fluid intake, elimination and gastrointestinal symptoms	3 (14)
		Support fluid and food intake	11 (52)
Eye disease	18 (22)	Observe changes in visual function and eye discomfort	0
Lung disease	17 (21)	Observe respiration, dyspnoea, cough and mucus	0
		Observe skin and cyanosis	0
		Observe fatigue	0
Skin disease and wounds	12 (15)	Observe changes in skin and wounds	3 (25)
		Perform skin and wound care according to prescription	11 (92)
Diabetes	11 (13)	Monitor blood glucose	6 (55)
		Observe and support food intake and eating habits	1 (9)
		Observe feet, prevent development of ulcers	0
Renal failure	11 (13)	Observe elimination of urine	1 (9)
		Monitor pulse and blood pressure	0
		Observe oedema, dyspnoea and weight	0
		Observe nausea, fluid intake and nutrition	0
Anxiety and depression	11 (13)	Observe changes in mental condition	4 (36)
		Observe ADL and nutrition	0
		Identify need of psychosocial stimulation	4 (36)
Neurological disease	10 (12)	Observe increase in neurological symptoms from actual disease	0
		Observe changes in ADL and provide support	0
		Observe bladder and bowel function	1 (10)
Cancer	10 (12)	Observe signs of changes in the affected organ	1 (10)
		Observe any new pain	1 (10)
		Observe nutrition	0
Medication use			
Cardiovascular drugs	60 (72)	Monitor pulse and blood pressure	6 (10)
		Assess orthostatic hypotension	0
		Observe skin and hydration	6 (10)
		Observe and treat oedema, dyspnoea and cough	6 (10)
Analgesics	53 (64)	Observe and treat pain	7 (13)
		Observe and treat constipation	2 (4)
		Assess fluid intake and need of extra fluid	1 (2)
		Observe nausea and appetite	3 (6)
		Stimulate physical activity	1 (2)
		Observe dizziness, unsteadiness and falls	2 (4)
Medication for stomach and intestinal disease	51 (61)	Observe nutrition, fluid intake, elimination and gastrointestinal symptoms	6 (12)
		Adjust fluid and food intake	14 (28)
Hypnotics	36 (43)	Assess sleep at night and daytime	0
		Observe activity at daytime	0
		Observe dizziness, unsteadiness and falls	0
		Assess long time need for hypnotics	0
Medication for respiratory organs	21 (25)	Observe respiration, dyspnoea, cough and mucus	0
		Observe skin and cyanosis	0
		Observe correct inhalation technique	1 (4)
Medication for anxiety and depression	17 (21)	Observe changes in mental condition	4 (24)
		Observe dizziness, unsteadiness and falls	0
		Observe fatigue and sleep at daytime	0
		Observe dry mouth and changes in nutrition	0
Antidiabetics	4 (5)	Monitor blood glucose	3 (75)
		Observe food intake	0

*ADL* Activities of daily living, *TMT* Trail making Test, *TUG* Timed Up-and-Go

^a^Needs are derived from three set of information: functional limitations, medication use, and diagnoses. The needs derived from these three information set will be partly overlapping. Accordingly, some interventions appear more than once. The denominator on each instance is the number of patients with the particular need due to the functional limitation, medication or diagnosis at stake

## Discussion

The patients showed a pronounced degree of frailty; more than one in three had severe limitations in mobility, eating and/or other ADL tasks, and more than seven out of ten exhibited impairments in psychomotor tempo, muscle function and/or focused attention. To our knowledge, frailty indicators in a population based sample of home-dwelling seniors receiving municipal services have not been studied in so much details before, though existing results accord with ours [2]. Although we aimed at including the most vulnerable home-dwelling elderly, as we had multimorbidity, daily home care and recent hospitalisation as inclusion criteria, for ethical reasons we had to exclude those who were terminally ill and those with too severe dementia as to be able to give a valid informed consent. The degree of observed frailty was nevertheless more pronounced than could be expected. Our findings emphasise a need for qualified observations and individually tailored pro-active treatment adjustments in order to maintain patients’ best possible health and function.

The level of frailty identified in this population may partly be explained by the Care Coordination Reform (CCR), which was implemented in Norway from 2012. The CCR gives the municipalities a greater responsibility for frail and sick elderly persons, and implies that services shall be provided in the people’s homes for as long as possible before moving the patient to a higher level of care. The reform also presupposes more individualized patient care and follow-up, demanding greater staff and budgetary resources allotted to the local authorities [21]. The reform has been successful in reducing the number of patients waiting in hospitals for available municipal care. However, this improvement may have been achieved on the cost of patients waiting longer at home for short as well as long term nursing home care [12]. As nursing home beds become an increasingly scarce resource, also in high-income Scandinavian countries, a growing percentage of very frail elderly persons will have to stay in their own homes and there be dependent on health care services from the municipality. Their frailty, multimorbidity and medical treatment indicate a need of qualified nursing interventions and cooperation with the general practitioner (GP) in addition to help with ADL. The degree of frailty and complexity found in our included patients raises the question whether these services are utilized in the most effective way as to cope with the increasing demands.

That the patients’ conditions deteriorated further over the eight month observation period, particularly with respect to mobility, is in line with results reported by others [22]. Recent interventional studies indicate that home based rehabilitation can be effective in home-dwelling elderly persons with functional impairments [23], but such measures seemed to be under-utilized in the districts we studied. Promising results also exist from interventions aimed at improved cooperation between hospitals and primary health care [10] and in identifying patients at particular risk of hospital readmittance [14]. In our opinion, a reasonable next step for model development studies should be to redirect the content of the routine health services provided at home more towards tailored observations of disease status, functional status, and drug effects in combination with rehabilitation, and with the health service input being flexible and open for day-to-day individualization as deemed appropriate by the health personnel themselves. Firm evidence for the effectiveness of such an approach is limited, but there are reasons to believe that it may be effective [24–28].

Despite the degree of frailty and the obvious need for qualified services, we found a marked discrepancy between the health services the patients received, and what the consensus group deemed necessary. The necessary health services recommended by the panel were all well within the competence base of skilled nursing practice. As pointed out by Olde-Rikkert and co-workers [29], nurses should have a key position in the day to day monitoring of the health state in frail patients, and in communicating with the GP when necessary. However, our results suggest that nurses only to a limited degree had been requested to provide assessments and evaluations in accordance with their professional competence, for example to pay particular attention to possible adverse drug effects, signs of uncontrolled symptoms, and potentially avoidable disease progression.

Based on the medication lists, many of the patients probably had heart failure, pain, constipation, sleeping problems or anxiety, but we did not find documentation in the home nurses’ patient record system describing the course of the health problems, or that effects of their pharmacological treatment had been systematically evaluated. Several patients used opioid analgesics, anxiolytics or hypnotic drugs posing a high risk of delirium, falls and gastrointestinal problems. It is likely that if the nurses to a larger degree were given the time, competence and opportunity to monitor important health indicators and coordinate medical follow-up when necessary, health and function of this frail patient group might be better maintained and hospitalisations avoided [14, 30]. This would, however, require an organisational model in which the nurses were authorized to adapt the services to the patients’ varying health needs from day to day, and were not bound to follow detailed service descriptions and schedules. Moreover, the nurses should collaborate closer with the patients’ GPs and have easy access to information on the patients’ actual diagnoses, the medication, and the indication for each drug given. It has earlier been pointed out that the multidose drug dispensing system, which is now being widely used in Norway as well as several other countries, seems to weaken each nurse’s responsibility for professional evaluations of the medication and its effect [31].

The patients’ complex health problems indicate that they are in need of specialised nursing competence. In particular, nurses caring for this group of patients should be trained in assessing nutrition, drug related problems, pain, ADL impairments and related problems. The training of home nurses in such issues seems to be insufficient in Norway [32, 33] as well as abroad [34], and continuity of care too fragmented to address such issues satisfactorily [35].

One might argue that the consensus group has been too comprehensive in defining the patients’ health care needs emerging from their diagnoses, medication and functional limitations. However, we aimed at a conservative approach in the consensus process, securing that interventions were only listed as necessary if all panellist agreed upon them. Moreover we aimed at identifying conditions that necessitated nursing interventions beyond assisting in ADL. Consequently, we will argue that the interventions listed constitute a minimum of what would be necessary to avoid preventable disease deterioration, functional decline and hospital readmittance.

Another objection might be that we have under-estimated the quality of the services that was in fact provided. An obvious weakness of our study is that we did not objectively record what was done in each individual encounter between patient and nurse. However, also for this question we used an approach actively favouring the present situation. If an issue was at all mentioned in the decision or the case notes, it was recorded as “done”. As health personnel working in the municipalities are instructed to comply ever closer to the formal decisions and the pre-specified time to carry them out, and at the same time ever more emphasis is put on documentation of what has been done on each patient encounter, we find it very unlikely that the needs we have formulated have been fulfilled to any substantial degree more than documented in the patients’ records.

A weakness of our study is the limited number of participants. However, to our knowledge, few if any other studies have recorded a comparable set of clinical data on a patient sample with comparable degree of frailty and functional and cognitive impairment. Moreover, a strength is the repeated assessment with validated and widely used functional instruments in combination with detailed information on disease state and medication use. We also consider the consensus panel approach to be a strength, enabling us to establish a reference standard for evaluation of the services provided.

## Conclusion

In conclusion, home dwelling elderly with multimorbidity who have been hospitalized the last year constitute a very frail group, and are prone to further functional deterioration. The patient group’s very diverse health care needs and unstable clinical condition suggest that for this group, resources for home based health care in the home should be used in a more flexible and pro-active way, aiming to prevent further functional decline and unnecessary hospitalization, and improve the patients’ symptom burden.

## Abbreviation

ADL: Activities of daily living
CCR: Care Coordination Reform
GP: General practitioner
REK: Regional Committee for Medical and Health Research Ethics
TMT: Trail making test
TUG: Timed up-and-go
